# A comprehensive analysis of the relationship between inflammasomes and autophagy in human tumors: Recent developments

**DOI:** 10.1002/ccs3.70035

**Published:** 2025-07-16

**Authors:** Sai Liu, Jingzhou Zhang

**Affiliations:** ^1^ Health Science Center Yangtze University Jingzhou Hubei China; ^2^ Faculty of Pharmaceutical Engineering University of N'Djamena N'Djamena Chad

**Keywords:** autophagy, immunity, inflammasomes, tumor

## Abstract

Autophagy and inflammasomes are essential cellular mechanisms that maintain homeostasis, regulate immune responses, and influence disease progression, especially in cancer. Autophagy, a lysosome‐mediated process, removes damaged organelles and misfolded proteins, allowing cells to adapt to stress. This involves autophagosome formation, fusion with lysosomes, and subsequent degradation of cellular cargo. In contrast, inflammasomes are multiprotein complexes of the innate immune system that detect pathogenic signals and cellular stress, initiating inflammatory cytokine release to facilitate tissue repair. Notably, both pathways play dual roles in cancer: Although they help preserve cellular integrity and suppress tumorigenesis, they may also promote tumor survival under adverse conditions. This review explores the molecular mechanisms underlying autophagy and inflammasome activity, emphasizing their complex interplay and regulatory networks within the tumor microenvironment.

## Graphical abstract

The interaction between inflammasomes and autophagy is crucial for maintaining the balance between necessary immune responses and the avoidance of excessive inflammation. Autophagy can inhibit the activation of the NLRP3 inflammasome by degrading endogenous activators, such as damaged mitochondria that produce reactive oxygen species (ROS), as well as by eliminating components of the inflammasome and associated cytokines. Additionally, the autophagic process plays a role in the unconventional secretion of IL‐1β, thereby influencing the inflammatory response. On the other hand, the activation of the NLRP3 inflammasome can also modulate the formation of autophagosomes through various mechanisms. This interplay between inflammasomes and autophagy is essential for orchestrating an appropriate host defense response while preventing detrimental inflammation.

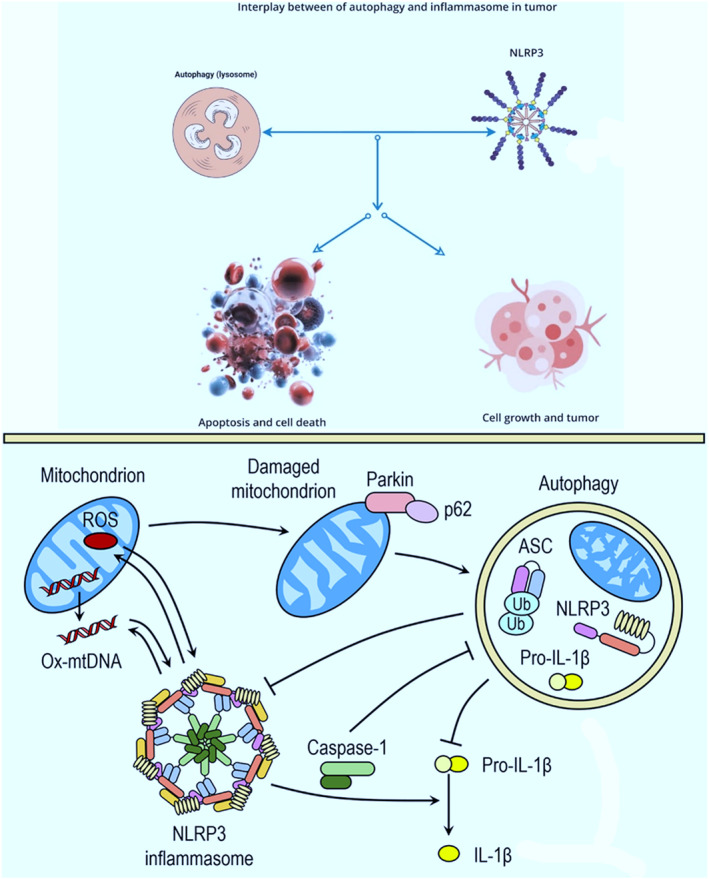

## 1 INTRODUCTION

Autophagy is an intracellular process that directs certain cellular components to the lysosome for degradation.^1^ Under normal, stable conditions, autophagy activity is low; however, when specific organelles are damaged, this system is activated, leading to the formation of autophagosomes that proceed with degradation. Research has shown that autophagy plays a critical role in various cellular events, including maintaining homeostasis, and also this process is closely related to human disease pathogenesis.^2^ Additionally, the autophagy process regulates cellular differentiation, cell death, and tumor formation.^3^ Inflammasomes, on the other hand, are protein complexes that initiate inflammatory responses to both external and internal pathological agents. They activate cytokines such as IL‐1β and IL‐18, which are essential for host defense against pathogens.^4^ Inflammasomes can also regulate autophagy within cells, thereby protecting cells from an excessive inflammatory response.^5^ Although prior studies have independently examined autophagy or inflammasome pathways in cancer, the current review offers a unique integrative perspective by simultaneously addressing the molecular crosstalk between autophagy, inflammasome activation, and noncoding RNA (ncRNA) regulation in tumor biology. This triadic interaction is often overlooked in existing literature. Here, we introduce the concept of a regulatory triangle involving autophagy, inflammasomes, and ncRNAs, and explore its implications across tumor progression, immune evasion, and resistance to therapy. This approach not only broadens our understanding of tumor immunometabolism but also provides a novel framework for identifying actionable targets in precision oncology.

## 2 AUTOPHAGY

**FIGURE 1:**
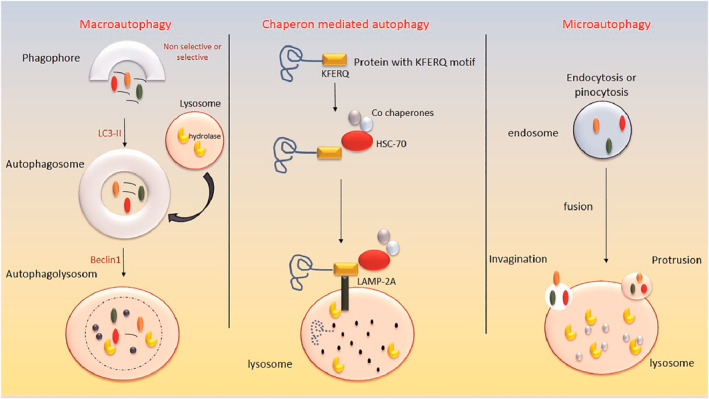
Overview of autophagy types. This figure illustrates the three principal types of autophagy: macroautophagy, chaperone‐mediated autophagy, and microautophagy. Macroautophagy, the primary form discussed in this review, is initiated by the formation of a phagophore, driven by LC3‐II. This structure matures into an autophagosome, which subsequently fuses with a lysosome via Beclin1‐mediated signaling to form an autolysosome that degrades cellular cargo. Chaperone‐mediated autophagy involves the recognition of specific protein substrates by HSC‐70 and their direct translocation into lysosomes via LAMP2A receptors. Microautophagy entails the direct engulfment of cytoplasmic components through membrane invaginations, followed by fusion with lysosomes for degradation.

In 1963, Christian de Duve used electron microscopy to identify various hydrolases, leading to the discovery of lysosomes and the explanation of autophagy as a cellular mechanism for breaking down organelles and proteins within the cytoplasm. He shared the Nobel Prize in Medicine with Albert Claude and George E. Palade in 1974.^6^, ^7^ Autophagy can be categorized into three main types: macroautophagy, chaperone‐mediated autophagy, and microautophagy. In macroautophagy, specialized double‐membrane vesicles called autophagosomes—originating from the rough endoplasmic reticulum (ER)—fuse with lysosomes to degrade proteins and organelles.^8^, ^9^ Chaperone‐mediated autophagy allows cells to selectively degrade specific proteins by tagging them with chaperone molecules, playing a role in regulating cellular metabolism by modifying lipids and glucose.^10^, ^11^ Microautophagy, on the other hand, degrades organelles and proteins by directly engulfing cellular components, impacting cellular survival^12^ (see Figure 1). Data suggest that autophagy declines with age, which may influence lifespan.^13^, ^14^ Another way to classify autophagy is based on nutrient availability: nonselective autophagy, which occurs during starvation, and selective autophagy, which occurs in nutrient‐rich conditions.^15^, ^16^ Forms of selective autophagy include mitophagy, ER‐phagy, xenophagy, and aggrephagy, which target mitochondria, the rough ER, pathogens, and proteins, respectively.^17^

Autophagy can be triggered by various cellular stresses, such as hypoxia, amino acid starvation, or a decrease in Adenosine Triphosphate (ATP), which initiates the regulation of specific genes needed for autophagosome formation. After forming, autophagosomes fuse with lysosomes, breaking down the target protein or organelle into smaller units.^18^ This review focuses on macroautophagy, and the term “autophagy” here primarily refers to this type of autophagy.

### 2.1 The mTORC1–ULK1–TFEB axis: Molecular control of autophagy in cancer and immunometabolism

Autophagy is a highly conserved lysosome‐dependent catabolic process essential for maintaining intracellular homeostasis, especially under conditions of metabolic stress.^19^ This tightly regulated mechanism involves the degradation and recycling of cytoplasmic components, including damaged organelles and misfolded proteins, thereby supporting energy balance and cellular survival.^20^ Among the critical regulatory nodes governing autophagy initiation and execution, the mTORC1–ULK1–TFEB signaling axis plays a central role in sensing environmental cues and coordinating appropriate autophagic responses.^20^, ^21^ Dysregulation of this pathway has been implicated in a wide spectrum of pathological conditions, notably cancer, neurodegeneration, and chronic inflammation.^22^

### 2.2 mTORC1 as a master regulator of autophagy

The mechanistic target of rapamycin complex 1 (mTORC1) integrates upstream signals from growth factors, amino acids, glucose availability, and oxidative status via the PI3K–AKT–Rheb signaling cascade. In nutrient‐rich conditions, mTORC1 phosphorylates and inactivates the ULK1–Atg13–FIP200–Atg101 complex,^23^ effectively suppressing the formation of phagophores—the initial membranous structures of autophagy.^24^ This suppression favors anabolic metabolism and biosynthesis but simultaneously impairs autophagic clearance of damaged mitochondria and aggregated proteins. In oncogenic contexts, constitutive mTORC1 activation exacerbates oxidative stress, supports tumor growth, and contributes to metabolic reprogramming.^25^

Upon energy deprivation, hypoxia, or reactive oxygen species (ROS) accumulation, AMP‐Activated Protein Kinase (AMPK) is activated and exerts dual effects: It phosphorylates ULK1 at activating residues while concurrently inhibiting mTORC1 through TSC2 and Raptor modulation.^25^ This leads to dephosphorylation and activation of the ULK1 complex, thereby initiating autophagosome biogenesis. In the tumor microenvironment (TME), this switch allows malignant cells to survive nutrient‐deprived and hypoxic niches, promoting therapy resistance and immune evasion.^26^

### 2.3 Mechanistic insights into autophagic progression

**FIGURE 2:**
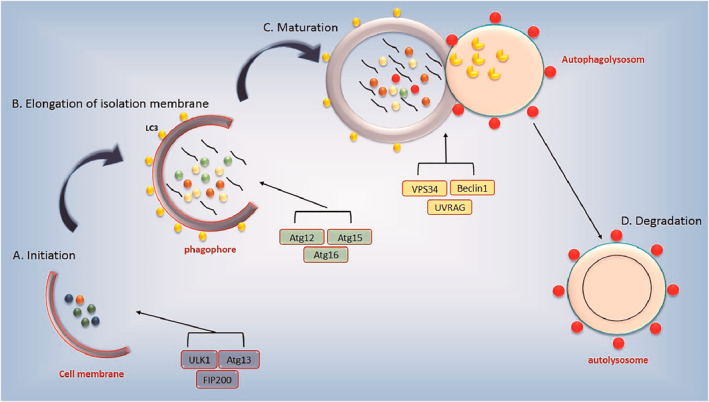
Sequential stages of macroautophagy. This schematic outlines the four major stages of the autophagic process: (A) Initiation: Triggered by the ULK1–Atg13–FIP200 complex, leading to the formation of the isolation membrane (phagophore). (B) Elongation: The Atg12–Atg5–Atg16 complex facilitates membrane elongation, whereas LC3‐II incorporation marks the developing autophagosome. (C) Maturation: The Vps34–Beclin1–UVRAG complex mediates autophagosome maturation and fusion with lysosomes to generate an autolysosome. (D) Degradation: Lysosomal hydrolases degrade the encapsulated cytoplasmic components, completing the autophagic turnover.

Autophagy progresses through four key stages: initiation, elongation, maturation, and degradation^27^ (Figure 2).

Initiation: Activated ULK1 is recruited to ER‐mitochondria contact sites, where it initiates phagophore formation.^28^ The class III PI3K complex I—comprising Vps34, Beclin1, Atg14L, and p150—catalyzes the local production of phosphatidylinositol 3‐phosphate (PI3P), facilitating the nucleation of PI3P‐enriched structures called omegasomes.^29^ DFCP1 binds PI3P and scaffolds the membrane expansion.^30^

Elongation: Two ubiquitin‐like conjugation systems regulate membrane elongation. The Atg12–Atg5–Atg16L1 complex is anchored on the outer phagophore membrane, stabilizing curvature.^31^, ^32^ Concurrently, LC3 (MAP1LC3) undergoes Atg4‐mediated cleavage and conjugation to phosphatidylethanolamine, forming LC3‐II, which incorporates into autophagosomal membranes and acts as a platform for cargo recognition via p62/SQSTM1, NDP52, and optineurin^33^. Rab32 and Rab33B facilitate membrane expansion and trafficking,^34^ whereas Jumpy (MTMR14) and MTMR3 antagonize PI3P signaling to prevent uncontrolled autophagy.^35^, ^36^

Maturation and fusion: Completed autophagosomes are transported along microtubules to the perinuclear region via the dynein‐dynactin complex. Rab7, together with the HOPS and SNARE complexes, mediates fusion with lysosomes to form autolysosomes.^37^, ^38^ This step is further modulated by the Ultraviolet Radiation Resistance Associated Gene (UVRAG)–Beclin1–Vps34 complex, which promotes lysosomal tethering and membrane fusion dynamics.^39^, ^40^

Degradation: Within the acidic lysosomal lumen, cathepsins and other hydrolases degrade sequestered material. The resulting monomers are recycled through lysosomal transporters for anabolic reuse. Declining levels of LC3‐II serve as a surrogate marker for active autophagic flux.^38^

### 2.4 Transcriptional reinforcement via TFEB

Transcriptional control of autophagy and lysosome biogenesis is orchestrated by TFEB, a member of the MiT/TFE family. Under basal conditions, TFEB is phosphorylated by active mTORC1 at Ser211, which promotes cytoplasmic retention via 14‐3‐3 binding.^41^, ^42^ Upon mTORC1 inhibition or lysosomal stress, TFEB undergoes calcineurin‐mediated dephosphorylation and translocates into the nucleus.^43^, ^44^ There, it binds to CLEAR (coordinated lysosomal expression and regulation) motifs in the promoters of autophagy‐related genes, including LC3B, LAMP1, CTSB, and ATP6V1G1, enhancing transcription of components critical for autophagosome formation and lysosomal function.^45^, ^46^

Importantly, TFEB‐driven lysosomal reprogramming has been linked to pathological contexts such as therapy resistance and immune modulation.^47^ In pancreatic and ovarian cancers, nuclear TFEB activation supports metabolic flexibility and alters inflammasome dynamics, leading to reduced immunogenicity and increased survival under cytotoxic stress.^48^ To provide a concise overview of this complex signaling cascade, Table 1 summarizes the molecular components and functional dynamics of the mTORC1–ULK1–TFEB axis, detailing their roles in autophagy and cancer metabolism.

**TABLE 1:** Key components and functional dynamics of the mTORC1–ULK1–TFEB axis in autophagy and cancer.

Component/stage	Molecular players	Key functions in autophagy	Implications in cancer/immunometabolism	References
1. Autophagy initiation	mTORC1, ULK1, Atg13, FIP200, AMPK	mTORC1 inhibits ULK1 in nutrient‐rich states; AMPK activates ULK1 under energy stress, triggering autophagy onset.	mTORC1 hyperactivation supports tumor growth; AMPK–ULK1 activation promotes survival in hypoxic TME.	19, 20, 21, 22, 23, 24, 25, 26
2. Nucleation	Beclin1, Vps34, Atg14L, p150, PI3P, DFCP1	Initiates phagophore formation at ER‐mitochondria contacts; PI3P promotes omegasome assembly	PI3K–Beclin1 complex dysfunction associated with defective autophagy and oxidative damage accumulation in tumors	28, 29, 30
3. Elongation and expansion	Atg12–Atg5–Atg16L1, LC3‐II, Rab32/33B, p62/SQSTM1, NDP52, optineurin, MTMR14, Jumpy	Conjugation of LC3‐II enables cargo selection and membrane extension; Rab proteins regulate trafficking; MTMRs dampen overactivation	LC3‐II/p62 dynamics correlate with tumor autophagic flux; mutations in LC3‐conjugation enzymes reported in aggressive tumors	31, 32, 33, 34, 35, 36
4. Maturation and fusion	Rab7, HOPS complex, SNAREs, dynein‐dynactin, UVRAG–Beclin1–Vps34	Autophagosome transport and fusion with lysosomes for cargo degradation	Fusion defects linked to chemoresistance; Rab7 and UVRAG alterations associated with poor outcomes in various cancers	37, 38, 39, 40
5. Lysosomal degradation	Cathepsins, proton pumps, LC3‐II turnover	Degradation of sequestered cargo and recycling of nutrients	Tumors exploit lysosomal recycling to fuel rapid growth; lysosomal enzyme expression linked to immunosuppression	38
6. Transcriptional regulation	TFEB, 14‐3‐3, calcineurin, CLEAR motifs, LAMP1, LC3B, ATP6V1G1	mTORC1 retains TFEB in the cytoplasm; inhibition triggers nuclear translocation and autophagy gene upregulation.	TFEB activation in pancreatic/ovarian cancer enhances metabolic plasticity, dampens inflammasome activity, and confers resistance	41, 42, 43, 44, 45, 46, 47, 48

### 2.5 Therapeutic targeting of the mTORC1–ULK1–TFEB axis

Therapeutic manipulation of this axis offers promising strategies in oncology and immunopathology. mTORC1 inhibitors, such as rapamycin and ATP‐competitive agents (e.g., Torin‐1), have demonstrated efficacy in reactivating autophagic flux and simultaneously reducing NLRP3 inflammasome activation by lowering mitochondrial ROS (mtROS) and dampening IL‐1β release.^49^, ^50^, ^51^ Such dual action not only restores intracellular proteostasis but also curbs proinflammatory signaling in the tumor microenvironment.^52^

Collectively, the mTORC1–ULK1–TFEB axis functions as a master regulatory module that governs autophagic homeostasis, coordinates cellular adaptation to stress, and modulates tumor–immune system interactions.^21^, ^53^ Given its centrality in both tumor biology and inflammation, this pathway represents a compelling target for next‐generation therapeutics aimed at restoring autophagy while controlling immune responses.

#### 2.5.1 Autophagy and tumors

In tumors, autophagy has a complex role that can vary depending on factors such as tumor stage, patient age, and cell type. Autophagy may either inhibit or promote tumor growth. Disruptions in autophagy are linked to various diseases in humans, including degenerative disorders and cancer.^54^, ^55^ Genetic studies in mice, involving genes such as *Atg7* and *beclin1*, have shown that when autophagy is impaired, tumor progression tends to increase.^56^ Based on these findings, it appears that autophagy loss can stimulate tumor growth; however, other reports indicate that active autophagy may also drive tumor invasiveness.^57^ One study on *beclin1* in breast cancer demonstrated that low levels of this gene are associated with a poor prognosis for HER2‐positive patients.^58^ When autophagy is disrupted, the resulting increase in ROS can lead to DNA damage and mitochondrial dysfunction, both of which contribute to tumorigenesis.^59^ In human breast cancer, *beclin1* expression is reduced, but reintroducing this protein can stimulate autophagy and inhibit tumor growth. For example, in women with breast cancer treated with tamoxifen, the drug was found to increase *beclin1* levels and activate autophagy.^60^

**FIGURE 3:**
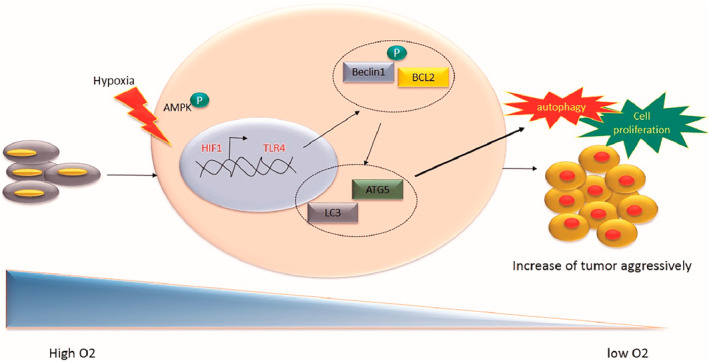
Hypoxia‐induced activation of autophagy in tumor cells. This figure illustrates the molecular events linking hypoxic stress to autophagy activation and tumor adaptation. Under low oxygen conditions, AMPK is activated and regulates downstream autophagy‐related genes. Concurrently, HIF‐1 enhances the transcription of TLR4, which facilitates the dissociation of Beclin1 from Bcl‐2. This cascade promotes the activation of LC3 and Atg5, initiating autophagosome formation and enabling cancer cell survival under hypoxic stress.

Many studies support the role of autophagy as a suppressor of tumor progression, though evidence suggests it can also promote tumorigenesis and treatment resistance in some cases.^61^ Data indicate that hypoxic conditions within tumors may initiate autophagy, promoting cell survival in these low‐oxygen regions^62^ (Figure 3). In the early phase of a tumor, autophagy can cause tumor suppression. However, in later stages, it often supports tumor growth and proliferation. At these advanced stages, autophagy plays a protective role by maintaining cellular health, controlling organelle and genome damage, and enhancing cell resistance to stresses such as hypoxia, nutrient starvation, and chemotherapy.^63^

#### 2.5.2 Inflammasomes

**FIGURE 4:**
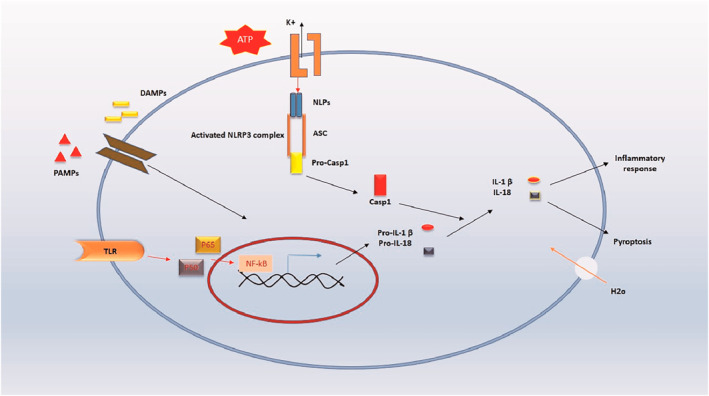
Canonical activation pathway of the NLRP3 inflammasome. This diagram summarizes the dual signaling pathways required for inflammasome activation and proinflammatory cytokine maturation. Signal 1 involves the recognition of DAMPs and PAMPs through pattern recognition receptors (e.g., TLRs), leading to NF‐κB activation and transcription of pro‐IL‐1β, pro‐IL‐18, and NLRP3. Signal 2, triggered by stimuli such as extracellular ATP, activates the NLRP3 inflammasome complex. This activation recruits and cleaves pro‐caspase‐1, which processes pro‐IL‐1β and pro‐IL‐18 into their mature forms. These cytokines mediate two primary outcomes: inflammation amplification and pyroptosis, a lytic form of programmed cell death characterized by membrane pore formation.

Inflammasomes are protein complexes in the innate immune system that detect infections and cellular changes, triggering responses to repair cell damage.^64^ They initiate this process by activating pro‐caspase‐1, which then facilitates the formation of pores in the plasma membrane, leading to an inflammatory cell death known as pyroptosis. Because of their role in cell death, any dysregulation in inflammasomes can contribute to autoimmune and metabolic diseases.^65^ The essential components of inflammasomes include a sensor protein, an adapter protein known as Apoptosis‐Associated Speck‐Like Protein Containing a CARD (ASC), and caspase‐1. Activation begins when the sensor protein undergoes conformational changes, which then activate caspase‐1. Caspase‐1 subsequently activates proinflammatory cytokines such as IL‐1β and IL‐18^66^ (Figure 4). Inflammasome assembly is triggered by detecting pathogen‐associated molecules and endogenous signals released from damaged organelles.^67^

Key inflammasome sensor proteins include NOD‐like receptors (NLRs), absent in melanoma 2 (AIM2), and pyrin. Among these, NLRP1, NLRP3, NLRC4, AIM2, and pyrin are well‐studied, although other sensor proteins have been proposed.^68^, ^69^ A hallmark of inflammasome activation is the assembly of sensor proteins, ASC, and caspase‐1 into a macromolecular complex, where caspase‐1 produces P10 and P20 through an autocatalytic process.^70^ In humans, NLRP1 is encoded by a single gene that includes a pyrin domain, function‐to‐find domain, and a Caspase Activation and Recruitment Domain (CARD). Anthrax lethal toxin can activate NLRP1, which leads to pore formation on the cell membrane, causing pyroptosis without caspase‐1 autolysis.^71^, ^72^ The most widely studied sensor, NLRP3, forms an inflammasome with ASC and caspase‐1 in response to a range of infections.^73^ NLRP3 activation occurs in two stages: Signal 1 induces NF‐κB activation and upregulation of NLRP3, IL‐1β, and IL‐18.^74^ Signal 2, triggered by extracellular stimuli such as bacteria, uric acid crystals, toxins, and viruses, activates NLRP3 to oligomerize with inflammasome components.^75^ Recently, protein kinase D has also been identified as an NLRP3 activator.^76^ Another inflammasome member, NLRC4, is activated by protein kinase C during Salmonella infection.^77^ Evidence shows that NLRC4 can combine with NLRP3, ASC, caspase‐1, and caspase‐8 to form inflammasomes in certain bacterial infections.^78^ AIM2 inflammasomes detect double‐stranded DNA in the cytosol during bacterial and viral infections. GBP2 and GBP5, interferon‐inducible proteins, are essential for AIM2 activation. IRGB10 is another factor that causes AIM2 activation. These proteins target bacterial membranes, releasing bacterial DNA into the cytosol, which AIM2 inflammasomes can then recognize.^79^ Interestingly, some anticancer drugs can damage the nuclear membrane, leading to DNA release and AIM2 inflammasome activation.^80^

#### 2.5.3 Inflammasomes and tumors

Studies show that in mice, a lack of NLRP3 can lead to colitis and colitis‐associated colorectal cancer (CRC) when treated with azoxymethan.^81^ NLRP3 promotes the secretion of IL‐18, a factor involved in repairing epithelial damage, which plays a protective role against colitis‐associated CRC.^82^, ^83^ Additionally, NLRP3‐mediated IL‐18 secretion may boost the antitumor activity of natural killer cells against colon cancer cells that have metastasized to the liver.^84^ Research indicates that ATP released by tumor cells treated with chemotherapy drugs activates NLRP3 inflammasomes and the IL‐1β receptor.^85^ Among NLR sensors, NLRP6 is another factor with antitumor properties, offering protection in tumorigenic conditions by activating caspase‐1 and promoting IL‐18 secretion in intestinal cells.^86^ The protective role of inflammasomes against tumors and cancer is not always linked to caspase‐1 and IL‐18; for example, research shows that NAIP1, a component of NLRC4, can prevent STAT3 activation and inhibit antiapoptotic gene expression.^87^ One study found that NLRC4 can suppress colorectal tumors by inhibiting cell proliferation and promoting cell death.^88^ In addition to NLR proteins, AIM2, a DNA‐sensing inflammasome, can prevent tumor formation via an inflammasome‐independent pathway by inhibiting stem cell proliferation and promoting cell death.^89^, ^90^

However, inflammasome activation can also promote tumor development.^91^ For instance, a mouse model study injecting B16‐F10 melanoma cells found that NLRP3‐deficient mice showed reduced lung metastasis compared to wild‐type mice.^92^ Similarly, in breast cancer research, inflammasomes and IL‐1β were found to promote tumor growth and migration.^93^ Obesity, often associated with a poor prognosis in breast cancer, has been linked to NLRC4 inflammasomes, which may accelerate cancer development and progression.^94^ In pancreatic adenocarcinoma, inhibiting NLRP3, ASC, and caspase‐1 was shown to reduce cell growth and metastasis.^64^ Researchers conclude that inflammasomes play varying roles in tumor dynamics depending on the specific cell type involved in the tumorigenic process.^95^ For example, inflammasome activation in colon cancer appears to protect epithelial cells from invasion. This dual behavior of inflammasomes suggests that tumor‐inhibitory strategies should be tailored according to the specific cell and tumor type involved.^96^

#### 2.5.4 Inflammasomes and autophagy interplay in tumors

Understanding the complex crosstalk between autophagy and inflammasomes has become increasingly important in the context of cancer biology. Rather than operating as independent processes, emerging evidence suggests that autophagy and inflammasomes are deeply interconnected through shared molecular pathways, such as mitochondrial dysfunction, ROS signaling, and ER stress.^97^, ^98^ This review proposes that their interplay forms a dynamic regulatory axis that shapes tumor behavior across multiple dimensions, including immune evasion, metabolic adaptation, and therapy resistance. Specifically, we argue that the balance or imbalance of this axis may determine whether the tumor microenvironment becomes pro‐survival or pro‐death. ncRNAs further integrate into this axis as upstream regulators and downstream effectors, linking autophagic and inflammatory responses with epigenetic control.^99^, ^100^ By synthesizing recent findings, we aim to present a conceptual model where the autophagy–inflammasome–ncRNA triangle is not only mechanistically central but also therapeutically actionable for personalized oncology. A critical regulator of both inflammasomes and autophagy in cancer is mitochondrial ROS. Disruption of autophagy can lead to oxidative stress and cell death, often linked to inflammasome activation.^101^, ^102^ Increased mtROS production can promote cancer cell survival and proliferation, as well as resistance to chemotherapy.^103^

One essential site for inflammasomes (specifically NLRP3) and autophagy activation is the mitochondrial‐associated membrane.^104^ Autophagy activation at MAMs can remove damaged mitochondria, preventing NLRP3 inflammasome activation and influencing tumor progression.^105^ MAMs also play a role in lipid and mitochondrial biosynthesis through a calcium (Ca^2+^)‐dependent pathway. In this process, Ca^2+^ transfer from the ER to mitochondria may damage the organelle and activate inflammasomes.^106^ Interestingly, IP3, a molecule located at MAMs, has antitumor effects, though its role in autophagy and inflammasome activation leading to cell death is not fully understood.^107^

PKR (double‐stranded RNA‐dependent protein kinase) can activate both autophagy and inflammasomes, releasing HMGB1, a protein with antitumor effects.^108^ However, PKR also interacts with eEF1A2 (translation elongation factor), promoting cancer cell survival.^109^ The UVRAG autophagy gene has an antitumor role, and its downregulation is associated with age‐related autophagy suppression and tumorigenesis.^110^ Immunity‐Related GTPase M, a GTPase involved in immunity, also plays a role in autophagy and negatively regulates NLRP3 activation by mediating autophagy of NLRP3 components.^111^

Studies reveal a connection between the autophagy and inflammasome pathways involving Atg7 and JNK2. Atg7 can activate autophagy in response to palmitic acid, increasing cathepsin B, which promotes IL‐1β secretion via NLRP3.^112^ Meanwhile, JNK2‐induced mitophagy under stress conditions prevents inflammasome activation. Research also shows that tumor‐derived autophagosomes can activate inflammasomes and stimulate an adaptive immune response against cancer cells.^113^ Mitophagy plays an important role in cancer prevention; for instance, Parkin translocation in chronic obstructive pulmonary disease (COPD) can cause mitochondrial dysfunction, leading to COPD‐associated cancers.^114^ PINK1‐mediated mitophagy can suppress pancreatic cancer through SLC25A28‐mediated autophagy. However, mitochondrial iron buildup can activate inflammasomes and promote tumor growth.^115^

Disruptions in autophagy can drive inflammasome‐mediated tumor progression through HMGB1 activation.^116^ The interplay between autophagy and inflammasomes affects the release of proinflammatory cytokines such as IL‐1β and IL‐18, which can activate inflammasomes. Disruption of this balance may contribute to CRC.^117^ In CRC, autophagy provides nutrient supply to support tumor cell proliferation, whereas inflammasome activation can lead to pyroptosis, a form of cell death. However, chronic inflammasome activation may facilitate tumor progression.^118^

In cancer, autophagy‐related cell death can activate ATP signaling pathways, leading to NLRP3 inflammasome activation.^119^ Autophagy also impacts APCs (antigen‐presenting cells) and can stimulate antitumor immunity, whereas inflammasomes influence proinflammatory cytokine production.^120^ Dysregulation in either process may affect tumor cell survival and prognosis. Recent findings highlight the roles of NLRP3 inflammasomes and mitophagy in CRC, affecting cell survival.^121^ Epigenetic factors, such as methylation, histone acetylation, and microRNA (miRNA), can also modulate autophagy gene expression, influencing inflammasome activity.^122^ The interplay between autophagy and inflammasomes in cancer is governed by a multitude of molecular regulators and signaling hubs. Table 2 provides a structured summary of the major pathways, cellular components, and their contributions to tumor behavior.

**TABLE 2:** Molecular crosstalk between autophagy and inflammasomes in cancer: mechanisms and functional outcomes.

Molecular component/axis	Interaction type	Effect on tumor biology	Mechanistic insight	References
Mitochondrial ROS (mtROS)	Activates inflammasomes, regulated by autophagy	Promotes cancer cell survival and chemoresistance	Damaged mitochondria generate ROS, leading to NLRP3 activation if not cleared by mitophagy.	101, 102, 103
MAMs (mitochondria‐ER contact)	Platform for both autophagy and NLRP3 activation	Regulates lipid metabolism, Ca^2+^ homeostasis, and tumor progression	Autophagy at MAMs prevents inflammasome hyperactivation and ER stress‐induced apoptosis	104, 105, 106, 107
PKR–HMGB1 axis	Dual activator of autophagy and inflammasomes	Antitumor via HMGB1 release; pro‐survival via eEF1A2 interaction	PKR triggers both HMGB1‐mediated immunity and tumor‐supporting translation via eEF1A2	108, 109
UVRAG (autophagy gene)	Autophagy inducer, inflammasome suppressor	Tumor suppressor	Downregulated in age‐related cancer models; controls phagosome maturation and inflammation	110
IRGM (GTPase)	Mediates autophagic degradation of inflammasomes	Suppresses inflammation and tumor progression	Targets NLRP3 for autophagic clearance	111
Atg7–JNK2–Cathepsin B axis	Autophagy–inflammasome bridge	Enhances IL‐1β secretion, affects inflammatory microenvironment	Atg7 increases cathepsin B; JNK2 supports mitophagy to prevent NLRP3 overactivation.	112, 113
Parkin/PINK1‐mediated mitophagy	Suppresses inflammasomes, maintains homeostasis	Tumor suppression in pancreatic and lung cancers	Parkin dysfunction leads to inflammasome overactivation; PINK1 controls iron and SLC25A28‐dependent mitophagy.	114, 115
HMGB1	Links autophagy disruption to NLRP3 activation	Promotes tumor progression and inflammation	Released during autophagy failure, activates inflammasomes	116
IL‐1β, IL‐18 (cytokines)	Inflammasome downstream products	May trigger pyroptosis or chronic inflammation	Autophagy regulates their release; imbalance leads to colorectal tumor progression.	117, 118
ATP–NLRP3 axis	Autophagy‐related cell death activates inflammasomes.	Immunogenic cell death and potential antitumor immunity	Autophagy enhances antigen release, but also promotes inflammasome priming.	119, 120
Epigenetic modulators (miRNA, methylation)	Regulate autophagy gene expression	Modulate inflammasome activity, affect prognosis	Alter transcription of Atg genes and inflammasome‐related proteins	122

#### 2.5.5 Autophagy and inflammasomes in disease

The studies reviewed highlight the critical role of regulating autophagy and suppressing inflammatory systems like the NLRP3 inflammasome in managing a wide range of diseases, including cancer, chronic inflammation, and degenerative conditions. The findings demonstrate that various interventions, such as SIRT3 overexpression, rapamycin, and YAP/TAZ inhibitors, can significantly enhance cellular and tissue health by restoring autophagic balance and reducing inflammation.

For instance, in doxorubicin‐induced cardiotoxicity, overexpression of SIRT3 improved cardiac function and reduced myocardial fibrosis by inhibiting the activation of NLRP3 and restoring autophagy through the mTOR/ULK1 pathway. Similarly, in inflammatory diseases such as colitis and lung injury, compounds such as lonicerin and NRF2 activators enhanced autophagy and mitigated oxidative stress, leading to decreased inflammation and tissue damage. In cancer therapy, targeting autophagy through chloroquine or Hydroxychloroquine sensitized tumor cells to chemotherapy and reduced tumor growth.

These findings underscore the therapeutic potential of modulating autophagy and inflammation. By targeting these pathways, it is possible to alleviate cellular damage, enhance immune regulation, and improve the efficacy of treatments in a variety of conditions, from inflammatory diseases to cancer and neurodegenerative disorders. This approach offers a promising direction for future therapeutic strategies aimed at improving patient outcomes in diverse pathological contexts (Table 3).

**TABLE 3:** Relationship of inflammasomes and autophagy in disease.

Cell/tissue type	Key mechanism	Treatment/intervention	Proposed mechanism	Key findings	Concentration/method	Citation
Heart (cardiotoxicity)	NLRP3 inflammasome via autophagy	SIRT3 overexpression	Inhibits NLRP3 inflammasome activation through the mTOR/ULK1 pathway	Reduced cardiotoxicity, improved cardiac function, and decreased myocardial fibrosis	Various doses (0.5, 1.0, 2.0 μM DOX)	123
Microglia (BV2)	NLRP3 inflammasome via ROS	Methylmercury (MeHg), Pien‐Tze‐Huang (PTH)	Induces inflammation through ROS; PTH inhibits NLRP3 inflammasome via AMPK/mTOR/ULK1	Increased inflammation and autophagy through ROS; reduced inflammation with PTH	0.5–2.0 μM MeHg; 0.05–0.15 mg/mL PTH	124, 125
Macrophages (THP‐1, BMDMs)	Mitophagy/NLRP3 inflammasome regulation	3‐MA, rapamycin	Autophagy inhibition activates the NLRP3 inflammasome; rapamycin enhances autophagy and reduces inflammation	Enhanced IL‐1β secretion with 3‐MA; reduced IL‐1β secretion with rapamycin	10 mM 3‐MA; 50 μg/mL rapamycin	126, 127, 128
Cancer cells (pancreatic, breast)	mTOR/autophagy	Chloroquine, HCQ, METTL14 knockdown	Inhibits autophagy flux to sensitize cells to chemotherapy	Increased chemotherapy sensitivity, reduced tumor growth	CQ: 10 μM, HCQ: 50 μM, siRNA knockdown	129, 130
Inflammatory diseases (gut, lung)	Autophagy/inflammasome regulation	Lonicerin, NRF2 activators (e.g., Dimethyl Fumarate (DMF))	Enhances autophagy, reduces ROS, and suppresses NLRP3 activation	Reduced inflammation and tissue damage; improved antioxidant response	3–30 mg/kg lonicerin; 30 mg/kg DMF	131, 132, 133
Neurons and Alzheimer's disease	PINK1/Parkin mitophagy, rapamycin	Rapamycin, PINK1/Parkin activators	Enhances mitophagy and autophagy to mitigate inflammation	Decreased inflammation and mitochondrial damage; improved neuronal survival	1 mg/kg/day rapamycin; 10 μM activator	133
Lung and ovarian cancer	YAP/TAZ‐autophagy‐NLRP3, DIRAS3/ULK1 pathways	YAP/TAZ inhibitors (verteporfin), RNF157‐AS1 knockdown	Suppresses tumor growth and immune evasion; enhances autophagy	Tumor growth and immune evasion reduced; improved chemotherapy sensitivity	5 μM verteporfin; 2 μM Diamminedichloroplatinum (DDP)	130, 134

#### 2.5.6 Relationship of autophagy, inflammasomes, and noncoding RNA

##### Noncoding RNAs as dual modulators of autophagy and inflammasome signaling in cancer

ncRNAs, particularly miRNAs and long noncoding RNAs (lncRNAs), are becoming increasingly acknowledged as critical post‐transcriptional orchestrators in the intricate landscape of gene regulation. They establish a critical nexus between the autophagy and inflammasome signaling pathways, which are intricately linked to cancer. These RNA molecules have a significant impact on tumor biology, as they fine‐tune inflammatory signals, reroute metabolic pathways, and shape cellular responses to stress.^135^, ^136^ Given that disruptions in ncRNA expression are directly implicated in phenomena such as immune system evasion by tumors, unchecked cancer progression, and the challenging reality of therapy resistance, they are compelling targets for novel therapeutic strategies.^137^, ^138^

A growing corpus of research is shedding light on the complex interplay between autophagy and inflammasome activity, which is regulated by miRNAs. For example, miR‐223 is a well‐documented miRNA that suppresses NLRP3 expression, thereby reducing the activation of the inflammasome and the consequent release of IL‐1β in tumor‐infiltrating macrophages and myeloid‐derived suppressor cells.^139^, ^140^ In a similar vein, miR‐495 alleviates chronic inflammation in the tumor's local environment by targeting both NLRP3 and caspase‐1, suggesting that miRNAs may have a broader ability to reduce inflammation that would otherwise foster tumor growth.^141^

The actions of miRNAs in relation to autophagy are not uniform; they can either facilitate or impede this process, with their functions fluctuating depending on the specific tumor environment and cellular background. This duality is evident in miR‐199a‐5p, which targets Beclin1 and reduces autophagy, thereby increasing the susceptibility of ovarian cancer cells to cisplatin.^142^, ^143^ On the other hand, when hypoxia induces metabolic stress, molecules such as miR‐301a and miR‐146a actually enhance the survival of tumor cells by increasing autophagy. This is achieved through either the inhibition of PTEN or the activation of AMPK.^144^, ^145^ Then there is miR‐34a, a miRNA that is particularly intriguing and has the potential to function as a dual‐action therapeutic agent by influencing both autophagy and cancer development through the SIRT1–mTOR signaling pathway.^146^, ^147^, ^148^

Conversely, certain lncRNAs, such as MEG3, frequently exhibit a reduced expression in a variety of malignancies, which implies that they serve as tumor suppressors.^149^ In fact, they can induce apoptosis and autophagy through p53‐associated mechanisms. However, the narrative is not straightforward, as the increased expression of other lncRNAs, including MALAT1 and BLACAT1, is associated with more aggressive tumor characteristics.^150^ Unfortunately, the survival of cancer cells and their resistance to chemotherapy are bolstered by the increase in LC3B and Atg7 levels, which is the mechanism by which these specific molecules activate autophagy.^151^ Another lncRNA with oncogenic potential, H19, follows a distinct approach by activating mTORC1 signaling. This action promotes cell proliferation while suppressing autophagy.^152^ HNF1A‐AS1 influences autophagy regulation by acting as a sponge for miR‐30b, which targets Bcl‐2, thereby affecting both resistance to apoptosis and autophagic potential, thereby adding another dimension to this complexity.^153^, ^154^

## 3 CONCLUSION

Autophagy, inflammasomes, and ncRNAs all work together in a complicated regulatory network that governs how tumors form, grow, avoid the immune system, and resist treatment. These pathways are important in cancer biology because they regulate cellular metabolism, inflammation, and survival through complicated feedback loops. A better understanding of how these systems come together, especially through important molecular pathways such as mTORC1–ULK1–TFEB and regulatory ncRNAs, has led to the discovery of possible approaches to intervene in a targeted fashion. From a translational point of view, using ncRNA‐based drugs to change autophagy or inflammasome activity is a good way to get around tumor resistance and make immunotherapy work better. For instance, bringing back the expression of tumor‐suppressive RNAs such as miR‐223 or MEG3 may reduce inflammation that promotes tumors while speeding up the process of autophagy. Blocking cancer‐causing RNAs such as MALAT1 or H19 may interfere with the ways that tumors stay alive through autophagy and make cancer cells more sensitive to traditional chemotherapies. We provide a conceptual model in which ncRNAs act as important nodes that control autophagy and inflammasome signaling in a way that depends on the situation. The sort and stage of the tumor, as well as microenvironmental elements such as low oxygen levels, immune cell infiltration, and metabolic stress, decide how much they affect regulation. The next round of studies must focus on three main areas:

1. **Functional characterization:** Using in vivo models to make the tissue‐specific and time‐dependent changes in ncRNA–autophagy–inflammasome interactions clearer.
2. **Therapeutic innovation:** Making safe and effective ways to distribute miRNA mimics, lncRNA inhibitors, and autophagy/pyroptosis modulators.
3. **Clinical translation:** Using multiomics data (transcriptomics, proteomics, and metabolomics) to group patients and tailor treatments for this trifecta.

The growing regulatory triangle of autophagy, inflammasomes, and ncRNAs is a promising base for new ways to treat cancer. In the future, researchers need to look at these interactions in specific tumor ecosystems to find biomarkers of response and resistance. This will help them make cancer treatments that are more accurate and last longer.

## AUTHOR CONTRIBUTIONS

**Sai Liu**: Conceptualization; writing—original draft; writing—review and editing. **Jingzhou Zhang**: Conceptualization; writing—original draft; writing—review and editing.

## CONFLICT OF INTEREST STATEMENT

The authors declare no conflicts of interest.

## ETHICS STATEMENT

Not applicable.

## CONSENT

Not applicable.

## CLINICAL CODE AVAILABILITY

Not applicable.

## Data Availability

Data sharing is not applicable to this article as no datasets were generated or analyzed during this study.
